# A Parallel Perifusion Slide From Glass for the Functional and Morphological Analysis of Pancreatic Islets

**DOI:** 10.3389/fbioe.2021.615639

**Published:** 2021-03-05

**Authors:** Torben Schulze, Kai Mattern, Per Erfle, Dennis Brüning, Stephan Scherneck, Andreas Dietzel, Ingo Rustenbeck

**Affiliations:** ^1^Institute of Pharmacology and Toxicology, Technische Universität Braunschweig, Braunschweig, Germany; ^2^Center of Pharmaceutical Engineering (PVZ), Technische Universität Braunschweig, Braunschweig, Germany; ^3^Institute of Microtechnology, Technische Universität Braunschweig, Braunschweig, Germany

**Keywords:** microfluidic perifusion system, borosilicate glass, femtosecond laser-structuring, islet of langerhans, calcium, insulin secretion, NAD(P)H- and FAD-autofluorescence

## Abstract

An islet-on-chip system in the form of a completely transparent microscope slide optically accessible from both sides was developed. It is made from laser-structured borosilicate glass and enables the parallel perifusion of five microchannels, each containing one islet precisely immobilized in a pyramidal well. The islets can be in inserted via separate loading windows above each pyramidal well. This design enables a gentle, fast and targeted insertion of the islets and a reliable retention in the well while at the same time permitting a sufficiently fast exchange of the media. In addition to the measurement of the hormone content in the fractionated efflux, parallel live cell imaging of the islet is possible. By programmable movement of the microscopic stage imaging of five wells can be performed. The current chip design ensures sufficient time resolution to characterize typical parameters of stimulus-secretion coupling. This was demonstrated by measuring the reaction of the islets to stimulation by glucose and potassium depolarization. After the perifusion experiment islets can be removed for further analysis. The live-dead assay of the removed islets confirmed that the process of insertion and removal was not detrimental to islet structure and viability. In conclusion, the present islet-on-chip design permits the practical implementation of parallel perifusion experiments on a single and easy to load glass slide. For each immobilized islet the correlation between secretion, signal transduction and morphology is possible. The slide concept allows the scale-up to even higher degrees of parallelization.

## Introduction

Pancreatic islets (islets of Langerhans) are functional mini-organs of the endocrine pancreas, containing multiple cell types. The most abundant cell type is the beta cell which makes up about two thirds of the total cell mass and which synthesizes and secretes insulin, the most important glucoregulatory hormone (Arrojo e Drigo et al., 2015; Sachs et al., 2020). Autoimmune killing of the beta cells causes type 1 diabetes (Daneman, 2006) and chronically progressive dysfunction of the beta cells is crucially involved in the pathogenesis of type 2 diabetes (Wang et al., 2013; Halban et al., 2014). Recently, further subgroup types have been established to improve individual treatment of the diabetic patient (Ahlqvist et al., 2018).

Stimulation of insulin secretion by high glucose requires its metabolic breakdown (Coore and Randle, 1964) leading to the increased generation of ATP by oxidative phosphorylation. The increased ATP generation closes K_*ATP*_ channels in the plasma membrane (Ashcroft and Rorsman, 1989; Henquin, 2000) and the resulting depolarization leads to Ca^2+^ influx via voltage-dependent Ca^2+^ channels and to Ca^2+^-triggered exocytosis of the insulin-containing granules (Gerber and Sudhof, 2002; Rorsman and Renström, 2003). In addition to triggering signals the beta cell metabolism of glucose and other nutrient stimuli generates signals which amplify the secretory response (Sato and Henquin, 2014; Schulze et al., 2017b). The nature of these signals and the mechanisms by which they increase Ca^2+^ -triggered insulin secretion are not fully understood (Prentki et al., 2013). It is therefore relevant to obtain coherent information on metabolic signaling and cytosolic Ca^2+^ concentration during insulin secretion, which is best studied by perifusion of isolated islets. In addition to basic research on stimulus secretion coupling, perifusion of pancreatic islets is a useful technique to investigate mechanisms of beta cell damage, to characterize the function of islets prior to transplantation or to study the mechanisms of insulinotropic drugs (Castiello et al., 2015; Ullsten et al., 2015).

In principle, perifusion of isolated primary pancreatic islets is an established *ex vivo* technique in a controlled environment to investigate the effect of physiological or pharmacological modifiers of insulin secretion (Panten et al., 1977; Alcazar and Buchwald, 2019). However, conventional perifusion (1) requires considerable amounts of islets per experiment, ranging from 50 to 250 or even 600 depending on the experimental set-up (Doliba et al., 2010; Schulze et al., 2017b), (2) uses comparatively large volumes of perifusion medium, which in turn may require large amounts of potentially expensive test compounds, and (3) severely restricts optical access to the islets due to the equipment typically used in the experiments, thereby limiting the number of parameters to the number of islet hormones which have been released into the perifusion medium or other inline analytics (Rountree et al., 2014).

In this perspective, the use of microfluidic perifusion devices designed as islet-on-chip systems offers clear advantages (Castiello et al., 2015). It (1) minimizes the amount of tissue per experiment (Dishinger et al., 2009; Bandak et al., 2018), (2) reduces the consumption of media and test compounds (Lenguito et al., 2017; Tao et al., 2019), and (3) makes tissue accessible to multi-parametric live-cell imaging to increase data coherence and experimental power, especially for fluorescent measurements (Nourmohammadzadeh et al., 2013; Schulze et al., 2017a). Moreover, (4) up-scaling which is in principle possible with microfluidic channel parallelization could permit a much higher throughput (Nourmohammadzadeh et al., 2016; Glieberman et al., 2019). Nevertheless, pancreas-on-chip systems made from glass described in the literature do not yet combine all these desirable features in a practical system for diabetes research. One reason for that is that these systems are only partially made from glass (Lu et al., 2018; Shik Mun et al., 2019). Monolithic glass chips allow for rapid prototyping, show low unselective drug absorption, low autofluorescence and high transparency (Piruska et al., 2005; Schwerter et al., 2016; van Meer et al., 2017). Furthermore, high thermal and chemical stability enable the repeated use of a monolithic glass systems (Hwang et al., 2019).

We present a 3D-structured perifusion slide entirely made from glass with five parallel microchannels to permit multiple independent experiments at the same time under a microscope. The system is scalable and allows for minimum tissue usage of one islet per experiment. It has the size of a conventional microscopic slide to enable the adaptation to microscopic measuring stands. Multi-parametric fluorescence live-cell imaging can be carried out as well as dynamic hormone secretion measurements. After the experiment, each islet is easily retrievable for histological examination to correlate the functional data with structural features. Due to its high chemical and thermal stability the perifusion slide can be reused.

## Materials and Methods

### Microdevice Design and Fabrication

The glass chip was fabricated from two structured Borofloat33 halves. The device consists of parallel microfluidic channels of the same length, cross section and with wells of identical dimensions. The channel width and height was set to 500 μm. Instead of a cubic well, an inverted pyramidal well with a basis of 500 × 500 μm and a depth of 500 μm was formed. The combined thickness of the both chip halves was 1,400 μm. The islet inlets in the upper half of the glass chip were covered by a standard 170 μm microscopy cover slip from borosilicate glass. The cover slip was not mechanically clamped to the glass chip but attached by carefully sliding the slip on the wet surface until interference colors appeared which sealed the islet insertion inlets at the usual pump rate of 40 μl per min in each well. The tubing was connected by a custom-made frame. The flow rate was generated by suction with a low-pulsation peristaltic pump (ismatec Cole-Parmer, Wertheim, Germany). This way, the light path of epifluorescence excitation conformed to standard conditions when an upright microscope was used.

The fabrication of glass system halves from 700 μm thick BOROFLOAT^®^ substrates (Schott AG, Mainz, Germany), was carried out in a laser workstation (Microstruct-C from 3D-Micromac, Germany) equipped with femtosecond YB:KGW laser (Pharos from Light Conversion, Vilnius, Lithuania) operated at the fundamental wavelength of 1030 nm. The beam was scanned over the substrate surface at 2,000 m/s and focused with an F-theta lens with a focal length of 100 mm. The laser was operated at a repetition rate of 600 kHz and emitted pulses of 215 fs with an energy of 14.65 μJ. These parameters led to an ablation depth of ∼50 μm. For layer-wise laser ablation the 3D-design was therefore converted into a stack of layers with 50 μm thickness. The areas to be removed were filled with scan lines with a distance of 4 μm starting at a distance of 4 μm from the desired contour edge. Each of these sets was rotated 30° against the previous one with a total of four sets per layer. For the subsequent layer the z position was decreased by 50 μm

After ablation, the system halves were cleaned in an ultrasonic bath filled with ethanol for 15 min, then immersed for 0.5 min in a solution of 45 ml H_2_O, 100 ml H_3_PO_4_ and 30 ml HF and finally subjected to a four-step program in a spray processor unit (Fairchild Convac, Neuenstadt, Germany). The spray processing routine began with a 6 bar high pressure distillate water spray and a simultaneous spinning speed of 5,500 rpm with a spin acceleration of 200 rpm/s^2^ for the duration of 60 s. For removal of organic residues a mixture of H_2_SO_4_ + H_2_O_2_ was sprayed on the substrate surface for a duration of 120 s while the spinning speed was reduced to 500 rpm with a deceleration of 200 rpm/s^2^. The substrate was rinsed with H_2_SO_4_ + H_2_O_2_ for 90 s while keeping the spin speed of 500 rpm. The cleaning process finished with dry spinning for 15 s at 550 rpm accelerated by 200 rpm/s^2^. To remove the H_2_SO_4_ + H_2_O_2_ residues from the surface, the substrates were manually rinsed in distilled water followed by dry spinning before alignment of both system halves in mask aligner (EVG 620 from EV Group, St. Florian am Inn, Austria).

A pre-bonding force was applied manually before the final thermal bonding was carried out in a muffle furnace at 630°C for 6 h with a pre-heating phase at 600°C for 15 min, while a force of 4 kN was uniformly applied to the entire 4” wafer surface. The combined thickness of both chip halves was 2,200 μm in the very first tests but reducing it to 1,400 μm permitted better yields of successful thermal bonding. After separation with a wafer saw (DAD320, Disco Corporation, Tokyo, Japan), the individual chips were rinsed with water. For use as support substrate another BOROFLOAT^®^ glass wafer (700 μm thick) was sputter-coated (LS 440 S, Ardenne Anlagentechnik GmbH, Dresden) on both sides with a gold layer of 300 nm. To avoid adhesions to the furnace bottom the chips were placed on this support wafer without contact to each other. They were dehydrated on a hotplate at 120°C for 5 min before they underwent a heat treatment at 730°C for 1 h in the muffle furnace. This heat treatment was performed twice to establish smooth glass surfaces within the micro channels (see results and discussion). Finally, the chips were removed manually from the support wafer with a sharp blade and gold residues on the chips were removed in a solution of 48 g I + 96 g KI + 960 ml H_2_O. Subsequently, the chips were cleaned in an ultrasonic bath filled with H_2_O for 15 min. Finally, the surface roughness was calculated as follows:

The *R_z_* value describes the maximum amplitude within the roughness profile

(1) $$R_{z}=R_{p}+R_{\nu }$$

Where *R_p_* is the maximum peak height and *R*_ν_ the maximum valley depth.

The Ra value is the arithmetic average value of the roughness profile

(2) $$R_{a}=\frac{1}{n}\sum_{i=1}^{n}\left|Y_{i}\right|$$

Where *Y*_*i*_ is is the height coordinate of the current observation and *n*the total number of observations.

### Microdevice Simulation

Three-dimensional CAD (computer-aided design) files of the microfluidic system with the immobilized islet were created using SolidWorks 2018 (Dassault Systèmes, Vélizy-Villacoublay, France) based on the geometries of the channel structures obtained from microfabricated devices using a 3D microscope (Keyence VHX series). The pancreatic islets were simplified as ideal spherical objects. The CAD files were imported to Ansys Fluent 18.0 (Ansys Fluent, ANSYS, Canonsburg, United States) for simulating the flow of two fluid phases. Krebs Ringer solution can be considered as exhibiting the characteristic properties of water at a temperature of 37°C (Kestin and Shankland, 1984). The simulation does not include diffusional transport, which is negligible compared to convection for the considered dimensions and time scales. The islets and the glass walls were assumed as non-deforming solid bodies. Of particular interest was the influence of the islet on the flow of the two phases during exchange. Furthermore, it was of interest to assess the shear stress exerted on the outer cell layer of the islet. For this investigation, twenty points were evenly distributed over the surface of the idealized islet. At these points the phase distribution, wall shear stress and pressure were obtained at each time step of the transient simulation. With the mean axial flow velocity in the microchannel υ_*m*_, the typical channel dimension *d* 500 μ*m* and the viscosity of water at 37 °C, ν the Reynolds numbers were calculated as:

(3) $$Re=\frac{\nu_{m}\cdot d}{v}$$

### Islet Isolation and Culture

Pancreatic islets were isolated from the pancreas of NMRI mice (12—14 weeks old, fed *ad libitum*) by a collagenase digestion technique and hand-picked under a stereomicroscope (Willenborg et al., 2012). In principle, freshly isolated and cultured islets can be used with this chip design. The smooth glass surface permitted an even flow of the fresh islets which are more sticky than cultured islets (Schulze et al., 2017a). For most of the experiments, cultured islets were used. Islets were cultured for up to 2 days in cell culture medium RPMI-1640 with 5 mM glucose and 10% fetal calf serum. Animal care was supervised by the regional authority (LAVES, Lower Saxony, Germany) and conformed to the current EU regulations.

### Microfluorimetric Measurements and Insulin Secretion

The microfluidic perifusion system was placed on the programmable stage (EK 75 x 50 Pilot, Märzhäuser, Wetzlar, Germany) of an upright Nikon Ni-E epifluorescence microscope (Nikon GmbH, Düsseldorf, Germany). Fluorescence was excited with a LED light source (Lumencor sola SM II Light engine) using the standard filter cubes for DAPI, GFP, and Texas Red. The fluorescence was collected by a Nikon S-Fluor objective (Plan Fluor 20x DIC N2, 0.5 N.A., WD 2.1) and images were recorded with a sCMOS camera (Nikon DS-Fi3). The imaging system was controlled by NIS-AR software and images were analyzed using the image processing software NIS-Elements (Nikon GmbH, Düsseldorf, Germany).

For the measurement of the cytosolic Ca^2+^ concentration ([Ca^2+^]_i_) using an upright epifluorescence microscope (Nikon Ni-E, Nikon GmbH, Düsseldorf, Germany), cultured islets were loaded with 5 μM Cal 630/AM (Biomol, Hamburg, Germany) and 0.04% pluronic acid by incubation for 60 min at 37 °C. Islets were then inserted in the micro device on the microscope stage and were perifused with HEPES-buffered Krebs-Ringer medium at 22 °C. The Cal 630 fluorescence was registered by using the Texas Red filter cube (exc. 562/40, dichroic 593, em. 624/40 nm), the NAD(P)H autofluorescence by the DAPI filter cube (exc. 350/50, dichroic 400, em. 460/50 nm) and the FAD autofluorescence by the GFP filter cube (exc. 470/40, dichroic 495, em. 525/50 nm). For the perifusion, an 8-channel peristaltic pump with low pulsation was used (ismatec Cole-Parmer, Wertheim, Germany), which was set at a pump rate of 40 μl/min per channel. For measurements on the stage of an inverted microscope (Zeiss Axiovert 135), cultured islets were incubated in Krebs-Ringer medium (5 mM glucose) with Fura-2 LeakRes (AM) at a concentration of 2 μM for 45 min at 37°C. The islets were immersed in a HEPES- buffered Krebs-Ringer medium, which was saturated with 95% O_2_ and 5% CO_2_. The fluorescence (excitation at 340 or 380 nm, dichroic separation at 400 nm, emission > 510 nm) was recorded with a cooled CCD camera (Pursuit, Diagnostics Instruments, Sterling Heights, MI, United States) and evaluated using Visiview software (Visitron, Munich, Germany).

For measurements of insulin secretion the parallel perifusion chip was thermostated by PT 100 sensors and heating foils located above and below the chip (Conrad Electronic SE, Germany). The fractionated efflux was collected in 96-well plates and the insulin content was determined by ultrasensitive-ELISA according to the manufacturer’s protocol (Mercodia, Uppsala, Sweden).

### Islet Morphology and Viability

The appearance of islets before insertion and after removal of the parallel perifusion slide was checked using transmitted light microscopy with DIC-contrast. Islets were placed in an open chamber with glass bottom and a water immersion objective (Zeiss Achroplan 40x, 0.75 w) was used for examination. Photomicrographs were taken by a Panasonic Lumix GX80 at the photo port of the upright Zeiss Axioscope FS microscope.

The live/dead assay was performed according to the manufacturer’s protocol (PromoCell, Heidelberg, Germany). The non-fluorescent membrane-permeable calcein AM-ester is cleaved in the cytosol of living cells whereby a green fluorescence is produced and retained within the cell when the plasma membrane is intact. Ethidium homodimer III is excluded from living cells but can reach the nucleus of dead cells. There it binds to nuclear DNA whereby the red fluorescence is about 40-fold intensified. After loading with the indicators, islets were placed on Petri dishes with glass bottom (ibidi GmbH, Gräfelfing, Germany) and either placed on an upright Nikon Ni-E epifluorescence microscope (Nikon GmbH, Düsseldorf, Germany) or on the stage of an inverted Nikon Ti2-E microscope fitted with a Yokogawa CSU W1 spinning disk unit. The green calcein fluorescence was excited at 491 nm, the red fluorescence of ethidium homodimer at 561 nm and collected by a Nikon CFI Plan Apochromat Lambda S40 XC Sil objective (40x, 1.25), which is designed to image thick specimen of living cells. Images were acquired by a sCMOS camera (Photometrics Prime BSI) under control of Visiview Premier software (Visitron Systems, Munich, Germany).

### Statistics

Results are presented as mean ± SEM. GraphPad Prism5 software (GraphPad, LaJolla, United States) was used for statistic calculations and non-linear curve fitting (ELISA).

## Results

### Chip Design and Assembly

**FIGURE 1:**
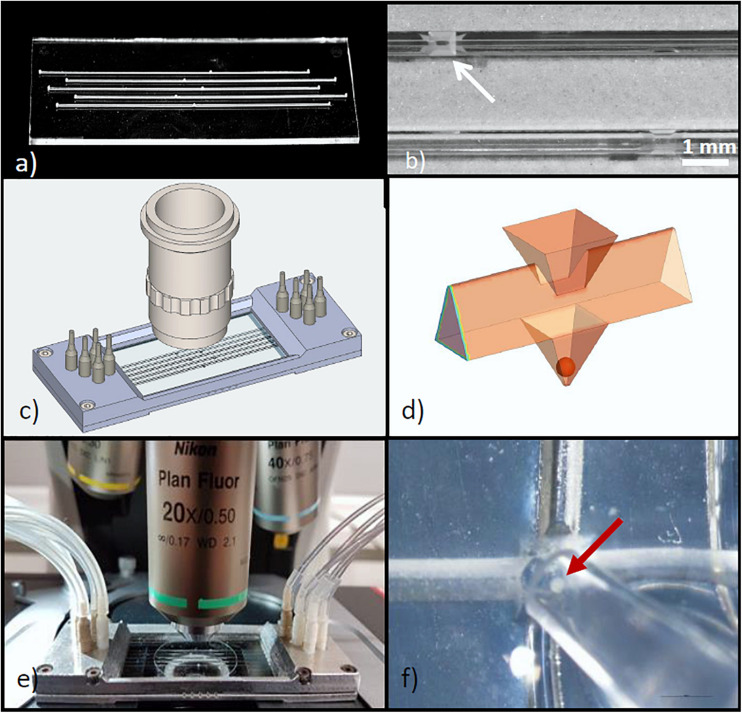
Design of the microfluidic system for parallel perifusion of single isolated islets. **(a)** View of the glass chip containing 5 parallel microchannels, each with a single well of 500 μm depth. The chip consists of 2 layers bonded together after structuring. The total thickness of the chip is 1.4 mm. **(b)** Detail of the chip surface, showing the inlet (arrow) through which the islet can be inserted into the well. **(c)** Isometric drawing of the parallel perifusion slide after attaching the glass chip to the metal frame. The metal frame holds PVC fittings to connect with the tubing. For size comparison a microscope objective is placed above the part where the wells are located. **(d)** 3D schematic view of the islet inlet, the microchannel shape and the pyramidal well below the inlet containing a spheroidal islet of 150 μm diameter. **(e)** Entire perifusion slide with attached tubing placed on the stage of an upright fluorescence microscope. The cover slip sealing the inlets is clearly visible. **(f)** A mouse islet (arrow) being inserted into the inlet above the well by use of an Eppendorf pipette.

The glass chip was fabricated from laser structured halves of 700 μm thick BOROFLOAT substrates, which give a 1,400 μm thick monolithic glass slide with five parallel microchannels (Figure 1a). The channel width and height of 500 μm of the preceding islet-on-chip design permitted a satisfactory velocity of medium exchange in the wells where the islets were located (Schulze et al., 2017a). However, the geometry of the wells in which the islets should be immobilized was changed. Instead of a cubic well, an inverted pyramidal well with a basis of 500 x 500 μm and a depth of 500 μm was formed to allow for a more precise and stable localization of the islets. Above each well an opening was cut by laser ablation in the upper half of the chip (in the glass capping layer). These openings are for the purpose of easy loading and unloading of the chip with islets using a pipette. Thus, a reliable positioning of exactly one islet in each well of the chip could be ensured (Figures 1b,f).

Since the required information on metabolic signals and cytosolic Ca^2+^ concentration ([Ca^2+^]_i_) during insulin secretion is recorded with a fluorescence microscope, the outer dimensions of the glass chip were adapted to those of a microscopic slide of 26 mm x 76 mm. With the symmetrical design either end of the parallel perifusion slide can be used as an inlet or outlet, which enhances the flexibility to adapt to diverse microscopic measuring stands. The requirement to perform microfluorimetric measurements led to a well position which can be easily accessed with a microscopic objective. As a result, upright but also inverted microscopes can be used to monitor the islet from the top and the bottom. To permit unobstructed use of high magnification objectives (40x and higher), the surface area of the glass chip must be large (Figure 1c). This requirement was even more critical in view of the intended sequential registration of the five wells which necessitates automated lateral and vertical movements of the microscope stage below the front lens of the objective.

**FIGURE 2:**
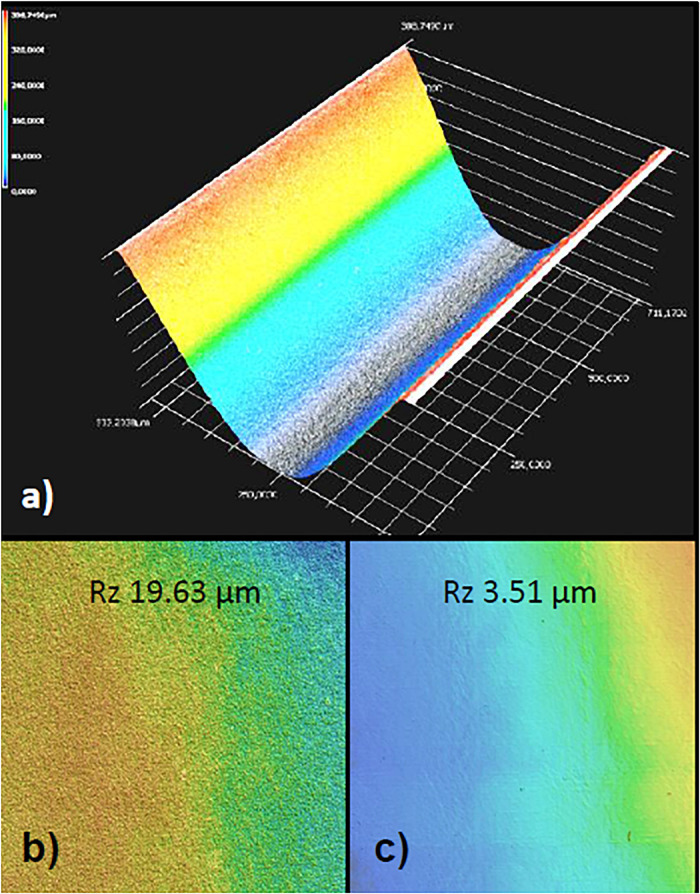
Laser structuring of borosilicate glass. **(a)** 3D image of the microchannel generated by depth measurements after the ablation process and the blow-off of debris. The channel is contained in the upper half of the parallel perifusion slide and is seen here from below. Note the nearly paraboloid shape which was intended to minimize turbulent flow in the channels. **(b)** Surface of the channel wall after wet cleaning procedure before bonding. Note the residual roughness of the glass surface as given by the Rz value of nearly 20. **(c)** Heating of the glass chip to just below the melting temperature reduced the surface roughness more than five-fold.

The volume of the pyramidal well is 0.0417 μl and the volume of a spherical mid-sized islet (150 μm diameter) makes up 4.25% of the volume of the well (Figure 1d). Each end of the microchannel was designed as a port, which opens to the upper surface of the glass chip. In cases where the experimental setup does not permit a direct loading of the islet via the inlets above the wells, an islet can also be loaded through these ports and then washed along the channel until trapped by the well. This requires microchannels with a smooth inner glass surface, guiding the islets to the individual wells (see also Figure 2). To connect the ports with tubing and a pump, a two-part mounting frame was designed (Figure 1c). The upper part contains screw threads suitable to connect the fittings for tubing and a groove for an o-ring, sealing the frame with the microfluidic device in the bottom part. The dimensions of the mounting frame were chosen to restrict the movement of the microscope stage under the microscope objective as little as possible.

The top of the assembled device was sealed with a conventional coverslip. Opening as well as the reopening was not impaired by the frame (Figure 1e). Filling of the microchannels by pumping in the Krebs-Ringer medium required covering the loading windows. After removal of the cover slip the islets were inserted using a standard Eppendorf pipette (Figure 1f). As long as no flow existed in the filled channels the islet descended smoothly into the pyramidal well. Loading of all five wells was completed within 1 min.

**FIGURE 3:**
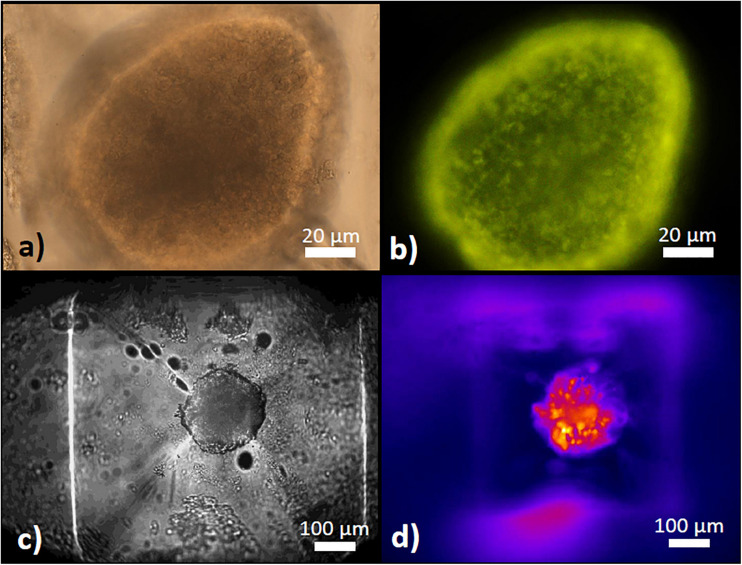
The pyramidal well for islet retention. **(a)** Appearance of a mouse islet within the well as seen from above with a 40x dry objective in transmitted light. **(b)** Appearance of the same islet as in **(a)** under epifluorescent excitation of TMRE fluorescence, labeling the mitochondria within the islet cells. **(c)** Appearance of a mouse islet within the well as seen from below with a 10x dry objective in transmitted light on an inverted microscope stage. **(d)** Appearance of the same islet as in **(c)** under epifluorescent excitation of Fura-2 AM fluorescence to determine [Ca^2+^]_i_ changes.

**FIGURE 4:**
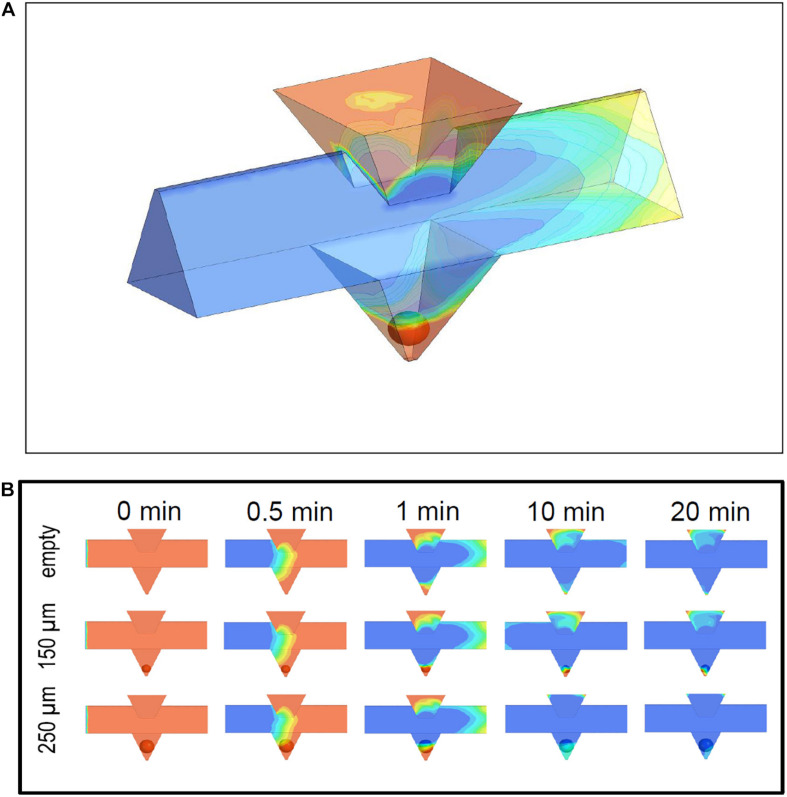
Simulation of medium exchange in the microfluidic system at 37°C. **(A)** 3D view of the microchannel and the pyramidal well containing an islet of 150 μm diameter. The time point is 1 min after the new medium (blue) has entered the microchannel and displaces the pre-existent medium (red). The upper part of the islet has just got into contact with the new medium. The pre-existent medium in the inlet is completely separated from the islet. **(B)** Time sequence of the fluid exchange, shown for the longitudinal section at the centreline of the microchannel and the well. The pre-existent medium (red) is displaced by the new medium (blue) at a pump rate of 40 μl per min. The time point 0 is set when the new medium is 1 mm away from the well. The upper row gives the results for the empty system, the middle row for an islet of 150 μm diameter, and the lower row for an islet of 250 μm diameter. The exchange velocity is influenced by the occupation of the well and by the size of the islet within the well.

While optical signals (fluorescence or absorption) can be registered through the lower half of the glass chip by using an inverted microscope (Figures 3c,d), a distortion-free image of the islet in the well was gained through the cover slip closing the inlets in the upper half (Figures 3a,b). For this reason the present islet-on-chip system is best used with upright microscopes. Here, the distance between the cover slip and the upper edge of the well is 700 μm, the distance between the cover slip and the upper edge of the islet ranges between 800 and 1,000 μm, depending on the diameter of the islet and the resulting position within the well (for a comparison between islets of 150 and 250 μm diameter see Figure 4B). Consequently, larger islets could be imaged with a 40x dry objective with working distance of 0.72 mm (Figures 3a,b), but smaller islets located further down the well required the use of objectives with a longer working distance (e.g., Plan Fluor 20x DIC N2, 0.5 N.A., working distance 2.1 mm).

The microchannels were generated by laser ablation in the upper half of the glass chip and the cross section of the microchannel was designed as triangular closed by the flat bottom of the lower half of the chip (Figure 2a). Particular care was taken to decrease the surface roughness after laser ablation. Heating of the bonded halves to just below the melting point reduced the surface roughness Rz by a factor of 5.6 and its arithmetic average *R_a_* by a factor of 3.43 (0.53 μm) (Figures 2b,c). As a result islets loaded through the window above the well were automatically centered in the well and did not stick to the channels or walls of the well.

### Simulation of Flows and Flow-Induced Forces

In the simulations (Figure 4A) the system was filled with the first phase (red), which is then displaced by the second phase (blue) inflowing with an initially sharp interface. While the volume of the loading window turned out to be a more slowly exchanging reservoir of the first fluid phase, the continuing slow wash-out of the first phase did not interfere with the fast exchange of the fluid phases in the well (Figure 4B). When the phase transition in the main channel has proceeded to about one millimeter behind the well position, the islet in the well comes into contact with the second phase. The medium exchange in the wells was influenced by the presence of islets. In particular, it was observed for small and medium-sized islets (up to 150 μm diameter) that the first fluid phase became temporarily trapped in the small volume below the islet (Figure 4B).

**FIGURE 5:**
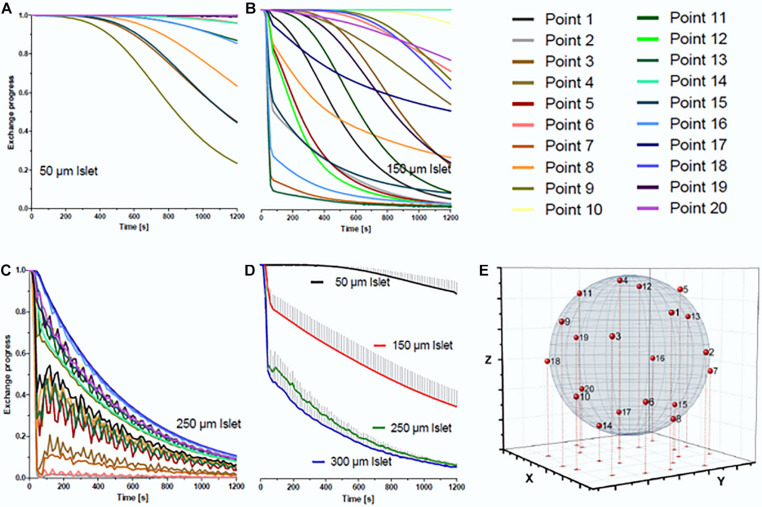
Simulation of the medium exchange at 37°C at the surface of islets of different diameters. For the simulation 20 points were evenly distributed over the surface of spheroidal islets of different diameter. The line color codes for the position of the point of simulated measurement, the color code is the same for all islets. The diagrams show the decrease of the pre-existent medium on the surface of **(A)** an islet of 50 μm diameter, **(B)** an islet of 150 μm diameter, and **(C)** an islet of 250 μm diameter. The time-dependent decrease of the mean value of all 20 measuring points is given in **(D)** for these three islets and, additionally, for an islet of 300 μm diameter. It becomes clear that the exchange process is substantially faster for islets of 250 μm than for those of 150 μm diameter, but does not accelerate much further with larger islets. The effect of diffusional exchange between the media was not included in the simulation. **(E)** Distribution of the 20 simulation points over the surface of an ideal spheroidal islet.

The influence of fluid trapping on the velocity of exchange of medium in direct contact with the islet was simulated at 20 points evenly distributed over the islet surface (Figure 5). Because small islets separate the volume underneath from the volume above, a large heterogeneity of the progression of the exchange is observed. For smaller islets having less total surface area the ratio of surface area exposed to the new phase to the non-exposed surface becomes smaller (Figures 5A,B). With larger islets (250 μm) (Figure 5C) a general biphasic behavior was discernible: a fast first phase which at some points led to a nearly complete exchange within 90 s followed by a slower exponential decrease. When considering the mean value of all 20 measurement points, 90 s of islet perifusion led to 20% phase one exchange of 150 μm islets and 50% of 250 μm islets. After 20 min the mean exchange of 150 μm islets was 63 and 92% of 250 μm islets (Figure 5D). In view of a fast exchange process leading to a homogeneous exposure, islets of 250 μm diameter should be preferentially used. Finally, the design of the loading window was vindicated by the result of the flow simulation. It was shown that this additional structure had no negative impact on the medium exchange in the well, because the fluid volume within this structure did not interfere with the fluid phase exchange in the islet surrounding (Figure 4A).

**FIGURE 6:**
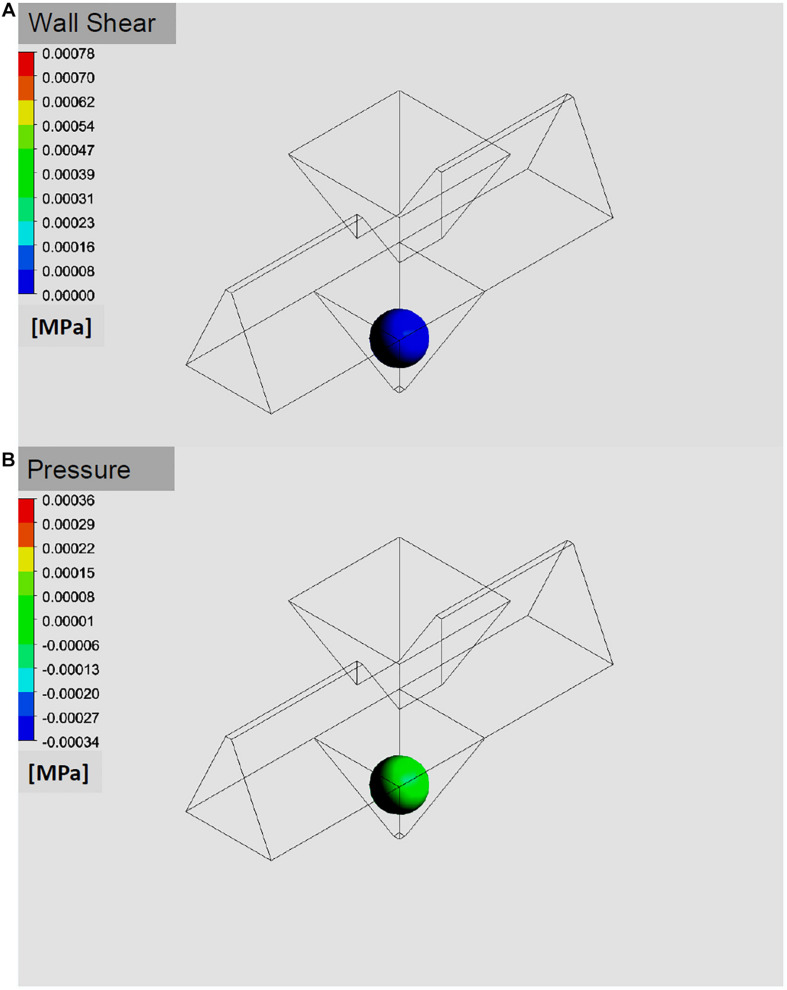
Simulation of pressure and shear stress exerted on an islet in the well. The benefit of a fast medium exchange has to be weighed against the forces exerted on the islet in the well. The simulation of pressure **(A)** and of shear stress **(B)** on the surface of a 150 μm islet suggests that only minimal mechanical force is exerted at the flow rates used for the simulations and the experiments (40 μl/min).

Both, the shear stress on the outer cell layer as well as the pressure acting on the islet surface were found to be below 10 Pa in the simulations (Figures 6A,B). For the volume flows used in the experiments purely laminar conditions at *Re* not exceeding a value of 3 could be confirmed. Uniformity of the channels was tested by flooding with fluorescent solution and washing it out again with bi-distilled H_2_O while recording fluorescence intensity with the CMOS camera of the upright microscope. The half-maximal fluorescence intensity was reached after 3 min, a steady state saturation was reached after 10 min. Upon wash-out, a 90% decrease was reached after 6 min. However, residual cellular and media debris from preceding experiments caused considerable variability in the maximal fluorescence levels of wash-in and out kinetics after 1 day of perifusion experiments. Overnight immersion in 10% HCl was sufficient to regenerate the chip.

### Multiparametic Measurements of Islet Function

**FIGURE 7:**
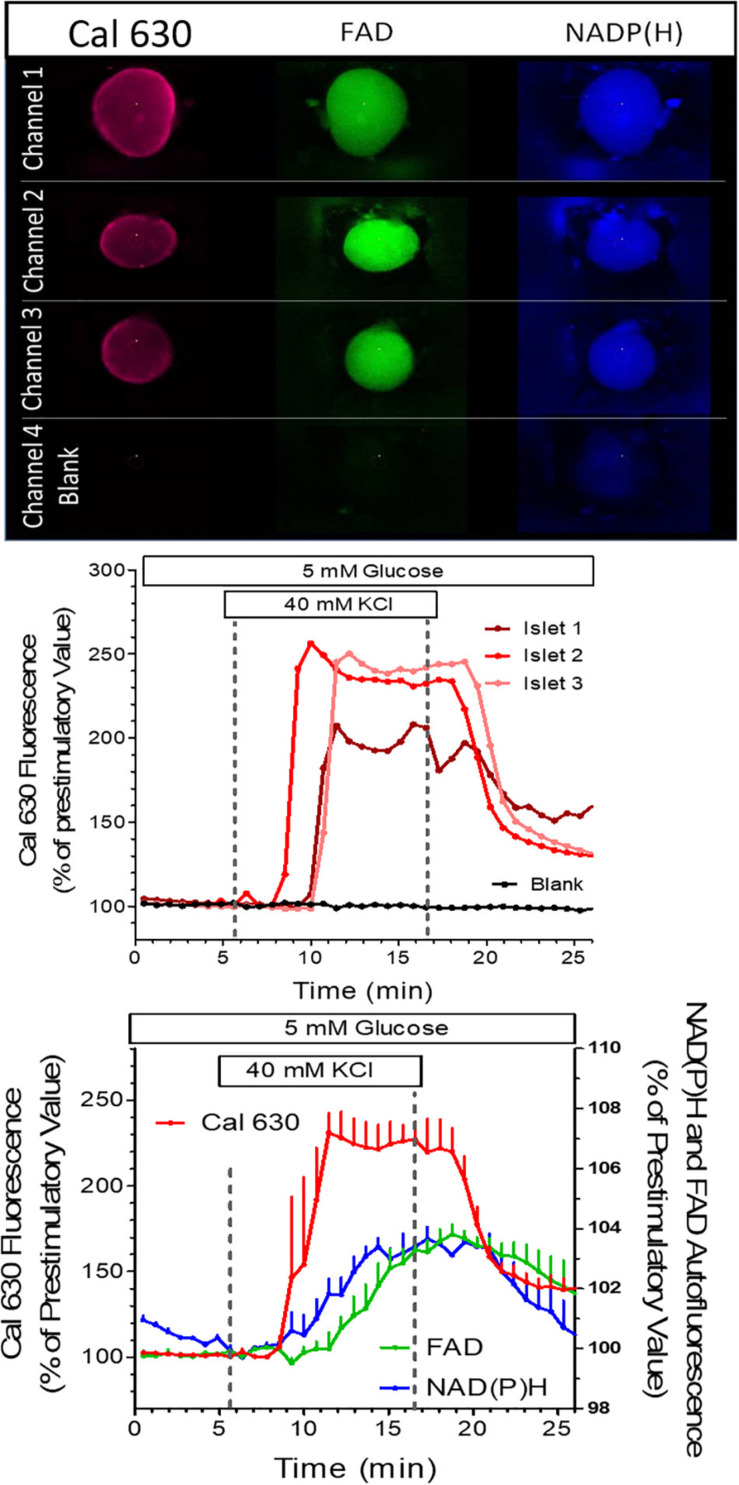
Simultaneous measurement in perifused islets of the NAD(P)H autofluorescence, the FAD autofluorescence and the fluorescence of the Ca^2+^ indicator Cal 630. Three islets of different size were loaded with Cal 630 AM, placed in wells, one each per well, and perifused with KR medium. A fourth well was left empty to provide a background signal. The wells were sequentially scanned, each islet being excited with three wavelengths to generate the dark blue NAD(P) H-, the green FAD-, and the red Cal 630 fluorescence. In the blank well a slight blue fluorescence was visible by cellular debris from a preceding experiment (upper panel). From 6 to 16 min the perifusion was switched to a medium containing 40 mM KCl. The intensity was normalized to the values at 6 min. The single traces of the Cal 630 fluorescence are shown in the middle graph, the mean values ± SEM of all three fluorescences are shown in the lower graph. Note the lack of fluorescence increase in the blank well and the increase of both NAD(P)H- and FAD-fluorescence during KCl depolarization.

In the first experiment the reaction of islets loaded with the fluorescent Ca^2+^ indicator Cal 630 AM to a strong potassium depolarization was tested. In addition to the fluorescence of the Ca^2+^ indicator the autofluorescence of NAD(P)H and FAD were registered (Figure 7, upper panel). These signals were compared with the background signal from an empty well. All islets showed the typical fast increase of the [Ca^2+^]_i_, whereas no change of signal intensity was recorded in the empty well. Thus, no spill-over of the fluorescence occurs (Figure 7, middle graph). This is important, since changes in autofluorescence were of much smaller extent. Also, they started slightly later than the increase in [Ca^2+^]_i_. and the modest but significant increase of NAD(P)H fluorescence was not accompanied by a decrease of the FAD fluorescence (Figure 7, lower graph). as would have been expected for nutrient stimulation of the islets. The faster onset of the [Ca^2+^]_i_, increase in channel 2 could traced back to a slight difference in tubing length, which emphasizes the need for precision not only during the chip production, but also during the experimentation.

**FIGURE 8:**
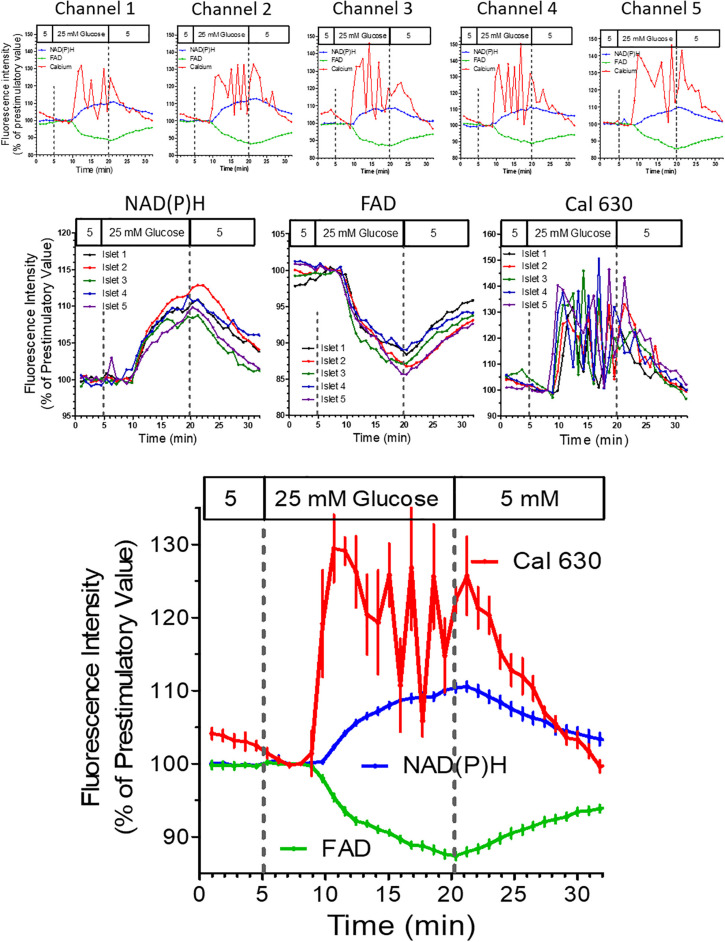
Multiparametric measurements in parallel perifused islets stimulated with glucose. Five islets were loaded with Cal 630 AM, placed in the wells, one each per well, and perifused with KR medium. From 5 to 20 min the glucose concentration of the perifusion medium was raised to 25 mM. The intensity was normalized to the values at 8 min (the time point when the medium reached the wells). The single fluorescence traces per islet are shown in the upper row, the single fluorescence traces per parameter are shown in the middle row. The lower graph shows the mean values ± SEM of the traces shown above. The onset of the oscillatory pattern of the cytosolic Ca^2+^ concentration (Cal 630 fluorescence) after 5 min contrasts with the continuous increase of the NAD(P)H-fluorescence and the continuous decrease of the FAD-fluorescence.

In the next experiment all five wells were filled with islets loaded with Cal 630 AM (Supplementary Material). Instead of a purely depolarizing stimulus a nutrient stimulation was performed by raising the glucose concentration from 5 to 25 mM (Figure 8). As in the preceding experiment the autofluorescences of NAD(P)H and FAD were registered in addition to the fluorescence of the Ca^2+^ indicator. Importantly, the pattern of the autofluorescence signals was similar with all islets and showed the typical opposite behavior of NAD(P)H and FAD in response to glucose stimulation. Interestingly, the increase of [Ca^2+^]_i_ showed an oscillatory behavior with considerable inter-islet heterogeneity (Figure 8, upper row).

However, the mean value of all five islets (Figure 8) gave a clear picture with a practically synchronous increase of [Ca^2+^]_i_ which after a reaching a peak value slowly decreased for about 5 min when an oscillation with an phase length of approximately 2.5 min set in. Upon wash-out basal [Ca^2+^]_i_ values were reached after 10 min, whereas the autofluorescences had not yet returned to prestimulatory values at this time point. The increase of NAD(P)H autofluorescence, but not of FAD-autofluorescence seems to lag behind the increase of [Ca^2+^]_i_ (Figure 8, lower graph), which is due to the low fluorescence excitation in the UV range.

**FIGURE 9:**
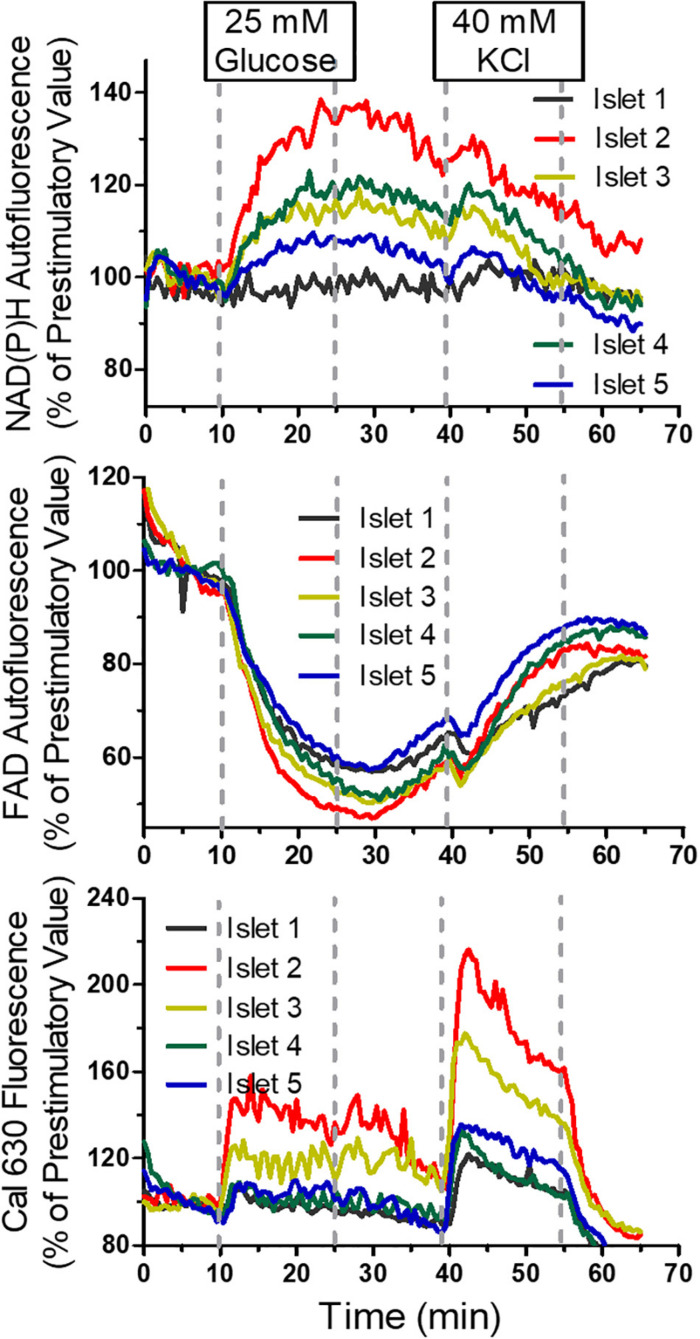
Multiparametric measurements in parallel perifused islets, sequentially stimulated with glucose and KCl. Five islets were loaded with Cal 630 AM, placed in the wells, one each per well, and perifused with KR medium containing 5 mM glucose. From 10 to 25 min the perifusion medium contained 25mM glucose and 40–55 min 40 mM KCl. The fluorescence intensities of NAD(P)H (upper panel), FAD (middle panel) and Cal 630 (lower panel) were normalized to the values at 5 min (the time point when the medium reached the wells). Note the inter-islet heterogeneity and the intra-islet homogeneity of the response to the stimuli. Interestingly, the FAD-response is less heterogeneous than the NAD(P)H-response. Only one islet shows Ca^2+^ oscillation.

Another experiment showed the large degree of inter-islet heterogeneity in response to the sequential stimulation with 25 mM glucose and, after return to basal glucose, with 40 mM KCl (Figure 9). The heterogeneity was most prominent with the NAD(P)H-fluorescence and least prominent with the FAD-fluorescence. However, the islet which showed the largest increase of the NAD(P)H fluorescence was also the one which showed the largest increase of the Cal 630 fluorescence and vice versa, the islet with practically no NAD(P)H increase was also the least responsive to raise [Ca^2+^]_i_. Thus, with regard to the parameters of stimulus-secretion coupling, there is a considerable degree of intra-islet homogeneity.

**FIGURE 10:**
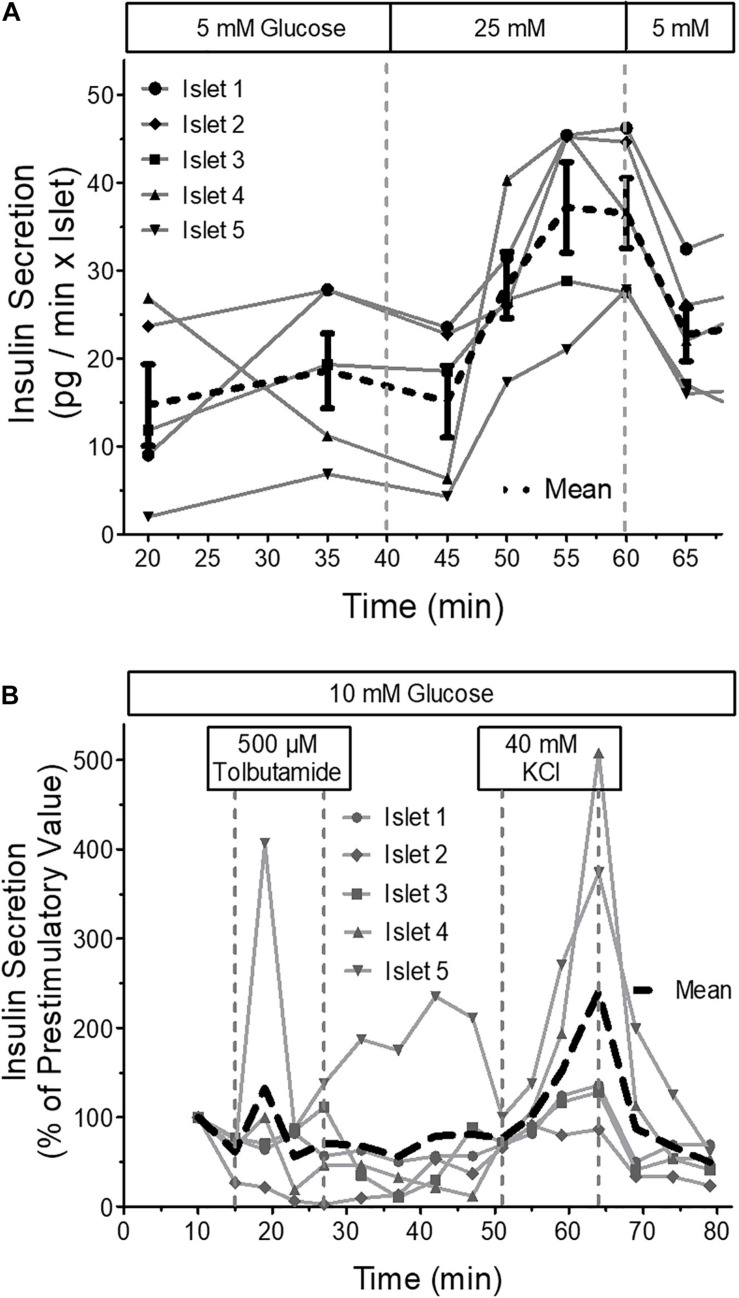
Insulin secretion of parallel perifused islets in the microfluidic system. Five islets of similar size were placed in the wells, one each per well, and perifused with KR medium. The efflux of the medium was collected by a 96 well plate and the insulin content of the fractions was determined by ultrasensitive ELISA. **(A)** From 40 to 60 min the glucose concentration of the perifusion medium was increased from 5 to 25mM glucose. Shown are the single values and the means of five independently perifused islets. Note the incomplete return to prestimulatory values during the wash-out of the glucose stimulus. **(B)** From 15 to 27 min the islets were stimulated with 500 μM tolbutamide and from 51 to 64 min with 40 mM KCl. Shown are the single values and the means of five independently perifused islets. This experiment was conducted at 22 °C. Still, there is a substantial increase of secretion in response to 40 mM KCl.

Measurement of insulin secretion at 37 °C and flow rates of 40 μl per min in each well showed that fractionated secretion measurements of a single perifused mouse islet is possible with a commercial ultrasensitive ELISA-Kit (Figure 10A). The marked temperature dependence of insulin secretion became visible in the following secretion measurement. Here purely depolarizing stimuli, the sulfonylurea tolbutamide and high extracellular potassium were used at 22°C (Figure 10B), which led to a modest transient increase by tolbutamide, but to a much more robust increase by 40 mM KCl, which was more sluggish than usual and continued until wash-out.

**FIGURE 11:**
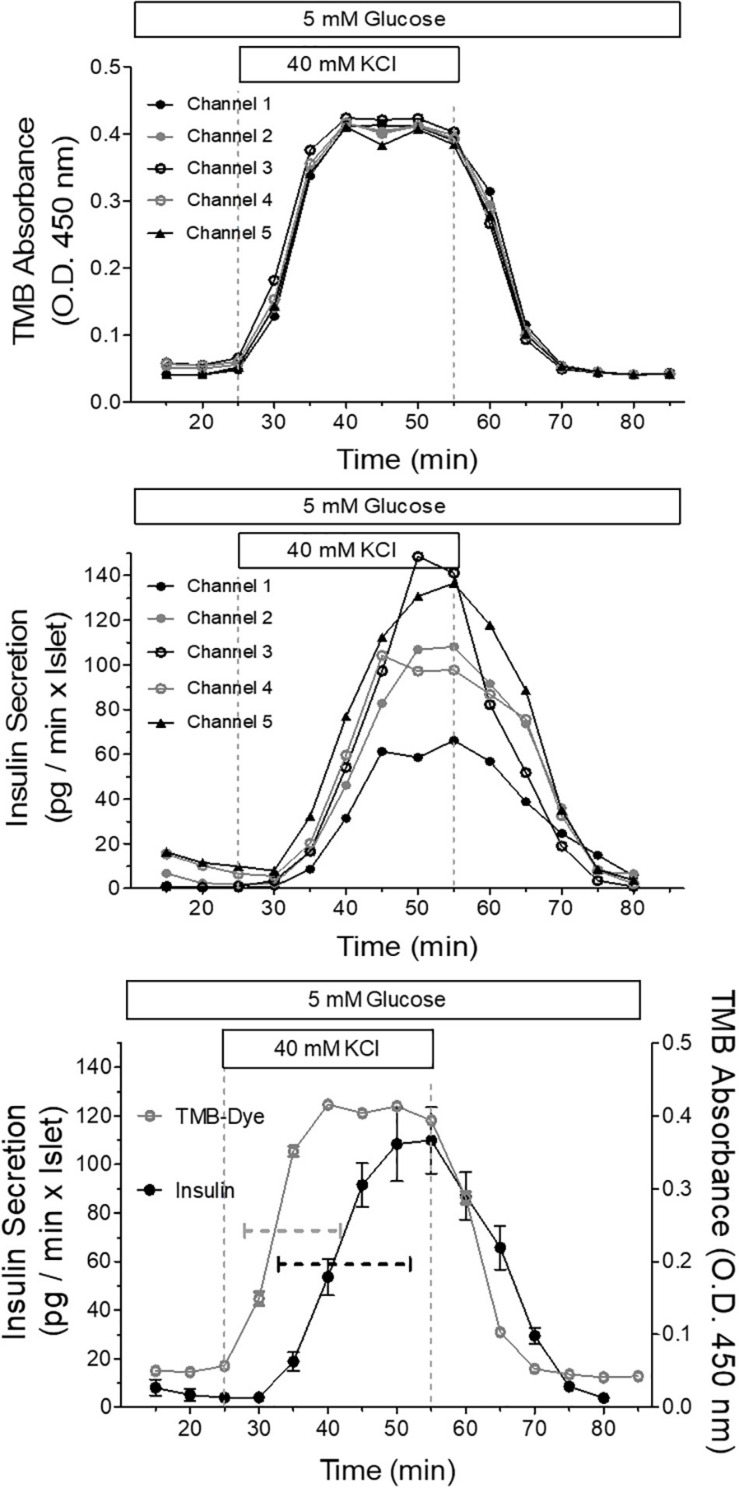
Relation between the stimulus pattern and the secretory response of parallel perifused islets in the microfluidic system. Five islets of similar size were placed in the wells, one each per well, and perifused with KR medium. The efflux of the medium was collected by a 96 well plate and the insulin content of the fractions was determined by ultrasensitive ELISA. From 25 to 55 min the islets were stimulated with 40mM KCl. This experiment was conducted at 37°C and each channel was perfused at a rate of 20 μl/min. Shown are the curves of five independently dye (TMB)-perfused channels to visualize the square wave stimulation by KCl (upper panel) and the secretion curves of five independently perifused islets responding to the square wave-stimulus (middle panel). The respective mean values ± SEM of secretion (closed circles) and dye perfusion (open circles) are depicted in the lower panel. The dashed lines indicate the time required from the onset of increase until steady state. When no SEM range is visible, it is smaller than the symbol size.

To demonstrate the relation between the stimulation pattern and the secretion pattern, which is inevitably influenced by the well shape, the increase of the extracellular K^+^ concentration from 5.4 to 40 mM was used as stimulus in all five channels (Figure 11). The moderate deviation of the KCl stimulus from a nominal square wave pattern was rendered visible by proportionally increasing the concentration of the dye tetramethylbenzidine (TMB) and measuring its absorption in the fractionated efflux, in the same way as was done with the secreted insulin. By depicting the single channels it becomes clear that the dye curves are practically identical (Figure 11, upper graph) whereas the single secretion curves show marked differences (Figure 11, middle graph). The comparison of the mean values of both curves showed that the secretory response followed the increase of KCl with a lag time of 5 min. The insulin secretion needed 19 min from onset to steady state, whereas the KCl stimulus needed 14 min. Upon wash-out prestimulatory KCl concentration was reached after 15 min whereas the insulin level needed 25 min (Figure 11 lower graph).

### Islet Recovery and Viability

**FIGURE 12:**
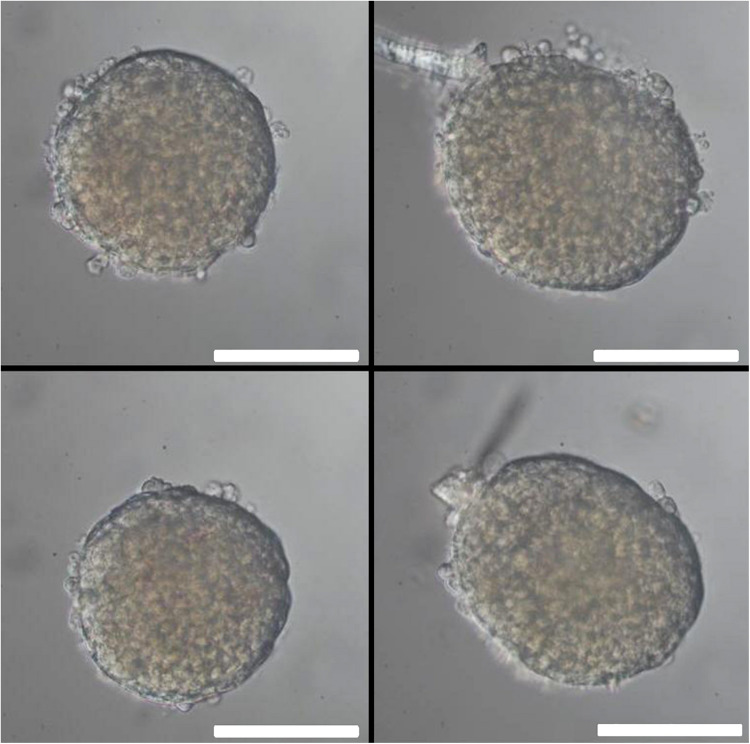
Islet morphology before and after removal from the microfluidic system. One day-cultured mouse islets were placed in the wells as shown in Figure 3a, removed by use of an Eppendorf pipette and inspected by microscopy. Upper row: islet of about 200μm (left) and 250μm (right) diameter before insertion into the well. Lower row: the same islets after removal from the microfluidic system. Note that the blood vessel remnant of the right islet has been shortened, but otherwise the islets appear intact. DIC-contrast, the length of the scale bar is 150 μm.

**FIGURE 13:**
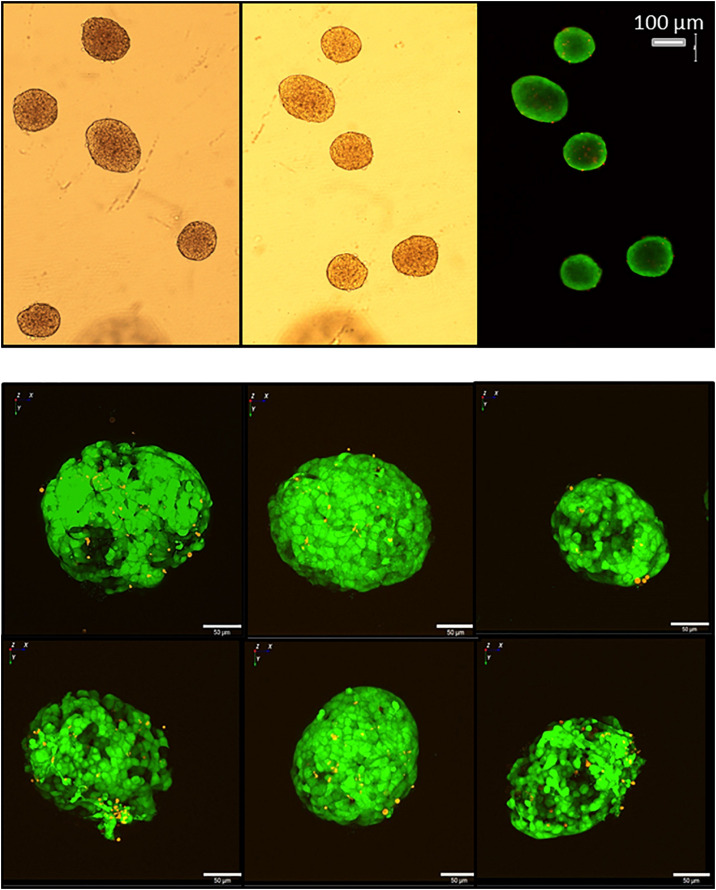
Live/dead assay of islets before and after removal from the microfluidic system. Upper micrographs: islets were inserted in and removed from the parallel perifusion slide and loaded thereafter with calcein (green fluorescence) and ethidium homodimer III (red fluorescence). Transmitted light micrograph before insertion (left) and transmitted light and fluorescence micrographs 5 min after removal from the chip (middle and right). Red nuclei are sparse, the entirety of the islets is labeled by calcein. Lower micrographs: calcein- and ethidium-loaded islets were imaged by spinning disk confocal microscopy before (upper row) and after insertion into the slide and removal (lower row). The number of red fluorescent nuclei indicating cell death did not increase and were only located in the islet periphery. The length of the scale bars is 50 μm.

The islet loading window also served the purpose of islet removal for further analysis. After pipetting five islets into the wells, followed by islet recovery after 5 min, the aspect of the removed islets was practically unchanged as compared to their appearance prior to the insertion into the wells (Figure 12). For more in-depth analysis the live-dead assay was used. With conventional epifluorescence a modest number of red fluorescent nuclei were observed in two of the islets (Figure 13 upper panel). This indicates the binding of ethidium which is excluded from intact cells. Since the fluorescent labeling was only performed after removal of the islets the test was repeated with labeling prior to insertion and removal. The analysis of these islets by spinning disc confocal laser scanning microscopy showed that the entire islet was loaded with the viability indicator calcein, that the ethidium fluorescence of the nuclei, when present, was localized at the islet periphery and that the number of fluorescent nuclei was not affected by the handling (Figure 13, lower panel).

## Discussion

The basic idea of the present islet-on-chip design was to fabricate a monolithic parallel glass chip, which is accessible from both sides. The chip was designed to permit multi-parametric measurements of dynamic intracellular processes and hormone secretion from single pancreatic islets contained in independently perfused parallel microchannels.

Even though cheaper manufacturing has made thermoplastic polymers like poly(methyl methacrylate) and polydimethylsiloxane (PDMS) widely used materials for organ-on-chip fabrication, glass chips still have important advantages, e.g., low autofluorescence, high transparency, low drug absorption, scalability for high throughput designs and reusability due to chemical and thermal resistance during repeated harsh cleaning processes. Furthermore, new processes to fabricate microfluidic channels from glass make this material attractive (Wang et al., 2018). Islet on chip systems made from glass have been successfully fabricated and applied to investigate islet physiology and hormone secretion (Dishinger et al., 2009; Lu et al., 2018). However, these systems were only partially made from glass and not ideally customized for parallel multi-parametric live-cell monitoring. The use of a monolithic glass slide should enable combination of advantages of microfluidic glass systems. While a range of glass materials have been used to fabricate microfluidic systems (Hwang et al., 2019), the above requirements were best met by the use of optical borosilicate glass (Borofloat). After structuring by a femtosecond laser, bonding of the two halves of a structured wafer gave the monolithic system with the size and robustness of a standard microscope slide.

Loading of the islet through a window above the desired point of the measurement has already been applied in different islet-on-chip systems (Dishinger et al., 2009; Bandak et al., 2018). However, here we sealed the system by a coverslip which sticks to the glass slide by capillary forces and pump suction. Therefore, no use of PDMS or adhesive foil with undesirable optical properties was necessary. This permitted inspection from above or from below with objectives of up to 40-fold magnification. However, with upright microscopes the small working distance of the 40x objective requires the use of large islets of about 250 μm diameter. Nevertheless, it was possible to monitor single cells within an islet using the brightfield or fluorescence mode.

Pyramidal well designs have been used to culture and test organoids (Kloß et al., 2008; Cha et al., 2017). We adapted this design to our glass microchannels for two reasons: Firstly, deep laser structured wells become at some point pyramidal-shaped due to laser beam deflection. Secondly, this shape will locate the islet automatically in the center of the observer window. For the latter process the glass surface has to be smooth, since the collagen fibers around the islets make them sticky. Since laser ablation leaves the glass surfaces roughened, problems with the islet loading occurred in an earlier chip design (Schulze et al., 2017a). Therefore we introduced a tempering step during fabrication to smoothen the glass surface. The degree of smoothening achieved corresponded to polishing (Kubies et al., 2011; Wang et al., 2018) and allowed the precise and easy loading of single islets into the wells.

Stable positioning of islets in the wells must meet two opposing requirements. On the one hand, the islet must be kept in a stable position throughout the experiment, on the other hand, the islet has to be effectively supplied with fresh media to enable the registration of cellular response dynamics. The measurement of wash-in and wash-out kinetics showed a satisfactory exchange inside the pyramidal well. Furthermore, the measurements showed that cleaning of the perifusion chip was necessary after each use with biological material, but that efficient cleaning was possible.

The flow simulations, which were carried out to test if the chip design meets the requirements, showed a clear correlation between the size of the islet in the well and the characteristics of the medium exchange. Bigger islet size was not only correlated with a faster but also with a more homogeneous exchange. Since only a minority of islets has a diameter of 250 μm or more it is relevant to note that upon downscaling of the pyramidal well the same characteristics of medium exchange can be expected with medium-sized islets. However, the homogeneity of exchange may not be of critical importance since the beta cells within the islet are electrically coupled by connexons, which leads to a high degree of synchronized responses (Cigliola et al., 2013; Farnsworth and Benninger, 2014). Furthermore, the exchange of the medium can also proceed via the extracellular space within the islet. Moreover, it has to be considered that in the small volume below the islet mass transfer by diffusion increases in relevance, which was not considered in the simulation model. Interestingly, the exchange characteristics were not affected by the islet inlet above the well because of the laminar flow of the media in the microchannels.

Thus, the results of the simulation can be regarded as providing a worst case-scenario of exchange velocity and exposure heterogeneity. The comparison of the secretion pattern in response to 40 mM KCl with the stimulus pattern, which was theoretically a square wave but was actually more similar to a steep sigmoidal saturation curve (see Figure 11), showed that the increase in secretion became visible after a lag time of 5 min and required ca. 35% more time than the stimulus to reach saturation. Upon wash-out the lag time was shorter but the additional time required for re-establishing the prestimulatory secretion was nearly 70% longer. This asymmetry points to the biological regulation of secretion as being responsible for at least part of the difference between the pattern of stimulation and the pattern of secretion. Also, the more heterogeneous pattern of insulin secretion as compared with the practically uniform pattern of the TMB absorbance, points in this direction. Taken together these data show that the insulin concentration pattern in the fractionated efflux, although somewhat deformed, represents the secretory activity sufficiently well. Clearly, the temporal resolution is inferior to systems specifically designed to achieve high resolution (Misun et al., 2020), but data such as presented in Figure 11 can be used to reconstruct the actual secretion profile for a given perifusion condition and thus to enable a meaningful correlation between the kinetics of the imaging data and that of the secretion data.

Mammalian cells are very vulnerable to mechanical forces, due to their thin outer plasma membrane (White and Frangos, 2007). In the body shear forces are usually less than 1 Pa but can in some regions reach up to 10 Pa, caused e.g., by cardiac output (Davies, 1995). Several groups have designed complex islet-on-chip systems in a way that flow-perifused islets were not exposed to more than mild shear stress (Silva et al., 2013; Glieberman et al., 2019). Positioning the islet in a well at the bottom of a microchannel also resulted in very small mechanical forces acting on it. Both the shear stress on the outer cell layer and the pressure acting on the islet surface were found to be below the critical 10 Pa in the simulations.

Dynamic monitoring of autofluorescence from redox equivalents like NADH, NADPH or FAD is a well-established method to investigate the mitochondrial metabolism of beta cells (Rocheleau et al., 2004; Panten et al., 2013; Schulze et al., 2017b). It is possible to simultaneously measure NAD(P)H and FAD (Schulze et al., 2017a) which can be combined with the measurement of fluorescent indicators of e.g., mitochondrial membrane potential or [Ca^2+^]_i_. Separate measurements of these parameters have already been performed with islet-on-chip systems (Dishinger et al., 2009; Lee et al., 2012; Bandak et al., 2018), but the simultaneous measurement of three parameters with five islets perifused in parallel has not yet been reported. Moreover, the relevance of high resolution imaging, capable to identify single cells during or after perifusion is emphasized by the recent findings that the heterogeneity of islet architecture has an impact on its functional state, e.g., proliferation and cell death (Benninger and Hodson, 2018; Dominguez-Gutierrez et al., 2019).

The first test of the suitability of the chip design for [Ca^2+^]_i_. measurements involved the stimulation of the islets by 40 mM KCl, since this strongly depolarizing concentration is known to induce a virtually immediate increase of the [Ca^2+^]_i_. While the increase was equally fast with all three islets, one islet responded earlier both upon wash-in and wash-out of the stimulus. The reason was found to be a slight difference in tubing length. This observation emphasizes that the precision of the perifusion is not only dependent on the chip design but also on the precise connection of the chip with the medium reservoir. This experiment confirmed that no cross-talk between the channels occurred and no spill-over of the strong fluorescence of the [Ca^2+^]_i_ indicator to the moderate autofluorescence occurred. A remarkable feature of the autofluorescence in this experiment was that its increase lagged behind the [Ca^2+^]_i_ increase and that the NAD(P)H increase was not accompanied by a FAD decrease but rather by a FAD increase. These features demonstrate that the changes in the mitochondrial function are secondary to the [Ca^2+^]_i_ increase and distinct from the changes induced by increased glucose concentration.

The metabolic stimulation was performed by concurrently raising the glucose concentration from 5 to 25 mM in all five channels. The increase of [Ca^2+^]_i_ was practically simultaneous in all five islets and after a constant elevation of [Ca^2+^]_i_ for 7 min the islets began to oscillate in phase until wash-out of the glucose stimulus. In contrast to the [Ca^2+^]_i_ neither the NAD(P)H- nor the FAD autofluorescence displayed an oscillatory pattern. While the [Ca^2+^]_i_ oscillation during glucose stimulation is a well-known phenomenon, the underlying mechanisms are still a matter of debate (Watts et al., 2014). Since activation of the mitochondrial metabolism precedes the increase of [Ca^2+^]_i_ it was somewhat puzzling that the increase of NAD(P)H autofluorescence lagged behind the increase of [Ca^2+^]_i_,. However, the glucose-induced decrease of the FAD-autofluorescence, a specific marker of mitochondrial activation (Panten and Ishida, 1975), occurred concurrently with the [Ca^2+^]_i_ increase. There may be a technical reason for the delayed increase of the NAD(P)H fluorescence. The signal intensity was by far the lowest, caused by the low excitation energy of the LED light source at wavelengths of 380 nm and below. Taken together, the experiments with KCl- and glucose-stimulation illustrate the relevance of multi-parametric measurements to obtain coherent data sets.

The small volume per islet ratio and the precise control of the pump rate allowed for the measurement of insulin secretion from a single islet by a commercial ultra-sensitive ELISA-Kit. The secretory response per islet is more heterogeneous than the [Ca^2+^]_i_ or autofluorescence response. Also, the time resolution is much more limited due to the fraction-wise determination of the insulin content of the perifusion medium. Additionally, the marked temperature dependence of insulin secretion (Schumacher et al., 2015) has to be taken into account, which is clearly visible by the sluggish response to KCl-depolarization at 22°C. However, warming the solution during transit from the reservoir to the chip increased the formation of air bubbles, which tend get stuck in the channels. So, to use the parallel perifusion slide to its full potential and to concurrently measure signal transduction and secretion from one islet, the fluorescence microscope has to be equipped with an environmental control chamber.

Post-perifusion analyses will give relevant information on morphological characteristics of functional beta cell adaption like number and distribution of secretory granules or on the occurrence of structural damage like mitochondrial swelling. Analysis of gene expression is another relevant application (Mulder, 2017; Essaouiba et al., 2020). Therefore, the parallel perifusion slide should enable the correlation between the functional data of the perifusion experiment and the morphological and molecular characteristics of islets. This requires the unmistakable identification of the islet and a quick removal at the end of the experiment to prevent secondary changes. This was made possible by the inlet above the well, which could also serve for islet removal and could easily be sealed by a standard borosilicate coverslip. The analysis of the islets by the fluorescent live/dead assay confirmed that loading into and removing from the wells did not affect viability or structure. Damaged cells, when present at all, were localized at the islet periphery and the number of fluorescent nuclei was not affected by the handling.

Regarding experimental scale-up, it has to be considered that collagenase digestion by injection into the bile duct regularly yields 100—150 islets within 1 h of manual work (Willenborg et al., 2012) and that among these about 50 islets of similar size can be found which should be suitable for loading into the parallel perifusion slide. Thus, upscaling by the factor of 10 would be possible for a higher throughput. However, one limiting factor is the parallel live cell imaging which requires a sufficiently long exposure time for weak fluorescence signals and at the same time should provide a sufficiently high time resolution. The time resolution achieved here was limited by the weak UV fluorescence excitation and the moderate sensitivity of the CMOS camera. Thus, with more dedicated live cell imaging equipment upscaling by a factor of 2 of 3 appears reasonably achievable without changing standard microscope set-ups. Likewise, the sensitivity of hormone measurement can be increased, to achieve a higher time resolution of the secretion pattern.

## Conclusion

To summarize, we have developed a 3D monolithic parallel perifusion slide compatible with upright and inverted microscopes, which permits to carry out five experiments simultaneously. The medium flow in the microchannels and islet-containing wells was characterized by simulations and fluorescence measurements and found to be adequate for the intended task. Cleaning after use is necessary and permits multiple uses. Islet loading was fast and unconstrained, as was the retrieval for further endpoint measurements. Consequently, dynamic multi-parametric live cell imaging and insulin secretion measurement was accomplished during parallel single islet monitoring. Further increase of the analytical power will come from the integration of additional sensors, e.g., miniaturized oxygen sensors or insertion of optical fibers for more specialized imaging or optogenetic techniques (Supplementary Material). The increased data coherence by multi-parametric measurements will widen our understanding of the function of the pancreatic islet. Upscaling is possible and will accelerate throughput for testing in the pharmaceutical industry, e.g., to identify promising drug candidates.

## Data Availability Statement

The raw data supporting the conclusions of this article will be made available by the authors, without undue reservation.

## Ethics Statement

Ethical review and approval was not required because the experiments on isolated islets were conducted *ex vivo* after sacrifice. Animal care in the central facility of the Technische Universität Braunschweig is supervised by the state authority (LAVES, Lower Saxony, Germany) and conforms to the current EU regulations.

## Author Contributions

AD, KM, IR, SS, and TS: conceptualization. DB, PE, KM, and TS: experimentation and visualization. AD, KM, and TS: methodology. IR and TS: writing – original draft. DB, AD, KM, IR, SS, and TS: writing – review and editing. All authors contributed to the article and approved the submitted version.

## Conflict of Interest

The authors declare that the research was conducted in the absence of any commercial or financial relationships that could be construed as a potential conflict of interest.
