# Supplementary material for: A Parallel Perifusion Slide From Glass for the Functional and Morphological Analysis of Pancreatic Islets
Source: Front Bioeng Biotechnol. 2021 Mar 5;9:615639. doi: 10.3389/fbioe.2021.615639 (PMC7982818; doi:10.3389/fbioe.2021.615639)
Supplement: Supplementary file 1 [file Data_Sheet_1.DOCX]

Supplementary Material

**A parallel perifusion slide from glass for the functional and morphological analysis of pancreatic islets**

Schulze T^1,3,*^, Mattern K^2,3,*^, Erfle P^2,3^, Brüning D^1,3^, Scherneck S^1,3^, Dietzel A^2,3^,

and Rustenbeck I^1,3^

^1^ Institute of Pharmacology and Toxicology, ^2^ Institute of Microtechnology, and ^3^ Center of Pharmaceutical Engineegring (PVZ), Technische Universität Braunschweig, D-38106 Braunschweig, Germany

**Supplementary Figure 1.** **Simultaneous measurement in perifused islets of the NAD(P)H autofluorescence, the FAD autofluorescence and the fluorescence of the Ca^2+^ indicator Cal 630 AM.** Five islets of different size were loaded with Cal 630 AM and inserted into the parallel wells of the channels. Subsequently islets were perifused with KR medium on the stage of an upright fluorescence microscope. The wells were monitored dynamically in x and y direction by programmable movement of the microscopic stage. Each islet was excited using three wavelengths to generate the dark blue NAD(P)H-, the green FAD-, and the red Cal 630 AM fluorescence. Corresponding graphs are shown in Fig 9.

b)


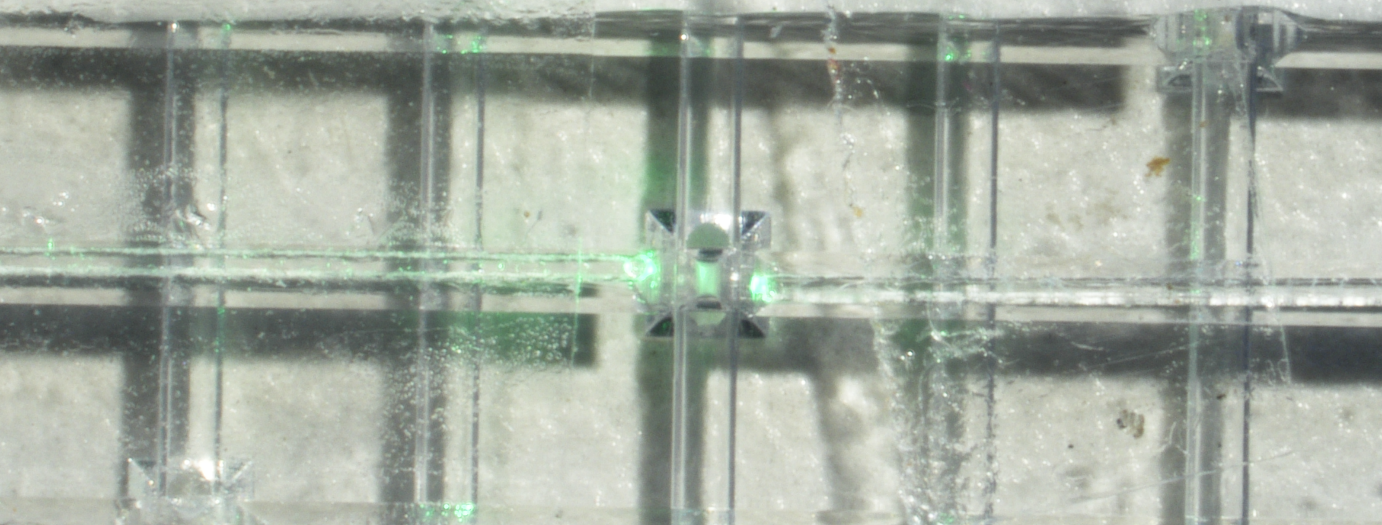

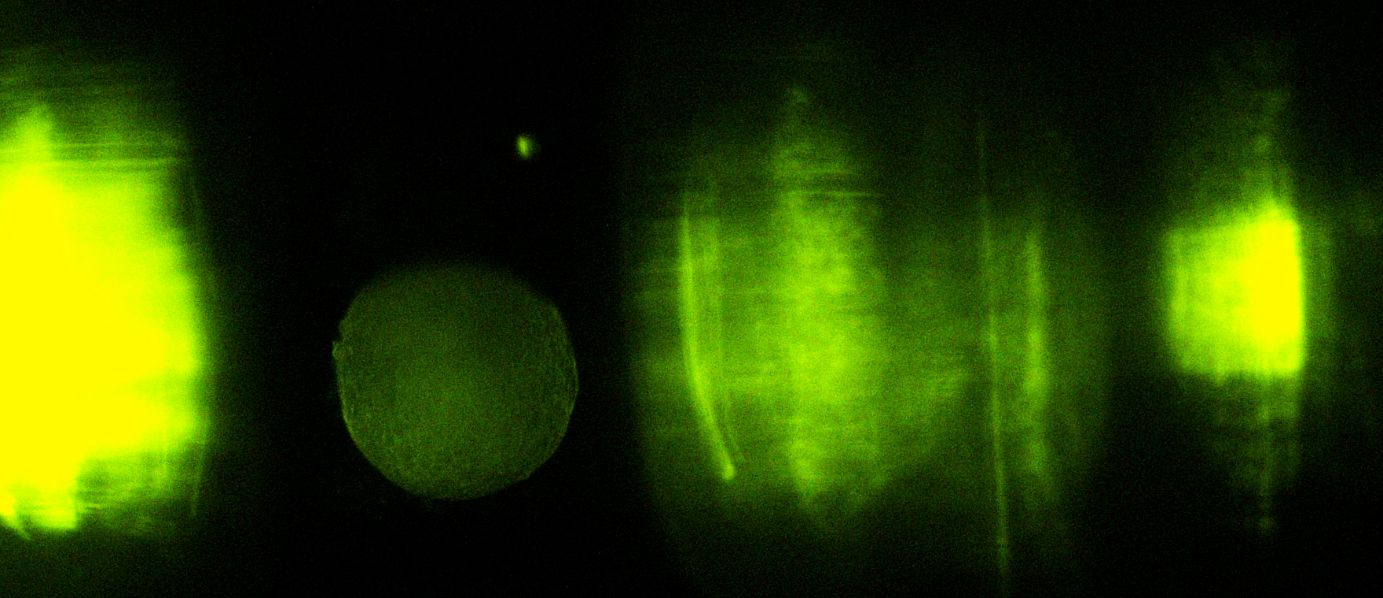


**Supplementary Figure 2.** **Integration of optical waveguides for independent illumination of wells and excitation of the centred pancreatic islet (a)** Alignment of two split wave guides with a diameter of 200 µm on the parallel glass chip. The wave guides are deployed in rectangular channels on the chip at the outer interface of the well and coupled to a green LED. **(b)** Appearance of an islet inside the well illuminated by waveguides coupled to a green LED. The independent light source is supposed to feature intended optogenetic applications.
